# Conversion of a Fleeting Open‐Shell Iron Nitride into an Iron Nitrosyl

**DOI:** 10.1002/anie.201908689

**Published:** 2019-10-22

**Authors:** Hao‐Ching Chang, Yen‐Hao Lin, Christophe Werlé, Frank Neese, Way‐Zen Lee, Eckhard Bill, Shengfa Ye

**Affiliations:** ^1^ Max-Planck-Institut für Kohlenforschung Kaiser-Wilhelm-Platz 1 45470 Mülheim an der Ruhr Germany; ^2^ Department of Chemistry National Taiwan Normal University 88, Ting-chou Rd. Sec. 4 11677 Taipei Taiwan; ^3^ Max-Planck-Institut für Chemische Energiekonversion Stiftstrasse 34–36 45470 Mülheim an der Ruhr Germany; ^4^ Department of Medicinal and Applied Chemistry Kaohsiung Medical University 100, Shi-Chuan 1st Rd. 807 Kaohsiung Taiwan

**Keywords:** EPR spectroscopy, iron, Mössbauer spectroscopy, nitrides, nitrosyl

## Abstract

Terminal metal nitrides have been proposed as key intermediates in a series of pivotal chemical transformations. However, exploring the chemical activity of transient tetragonal iron(V) nitrides is largely impeded by their facile dimerization in fluid solutions. Herein, in situ EPR and Mössbauer investigations are presented of unprecedented oxygenation of a paramagnetic iron(V) nitrido intermediate, [Fe^V^N(cyclam‐ac)]^+^ (**2**, cyclam‐ac^−^=1,4,8,11‐tetraazacyclotetradecane‐1‐acetate anion), yielding an iron nitrosyl complex, [Fe(NO)(cyclam‐ac)]^+^ (**3**). Further theoretical studies suggest that during the reaction a closed‐shell singlet O atom is transferred to **2**. Consequently, the N−O bond formation does not follow a radical coupling mechanism proposed for the N−N bond formation but is accomplished by three mutual electron‐transfer pathways between **2** and the O atom donor, thanks to the ambiphilic nature of **2**.

## Graphical abstract

**In situ EPR and Mössbauer** investigations reveal unprecedented oxygenation of a transient paramagnetic iron(V) nitrido intermediate yielding an iron nitrosyl species. Computational studies suggest that the N−O bond formation involves transfer of a formally closed‐shell singlet O atom to the (FeN)^2+^ core concurrent with synergetic orbital interactions.

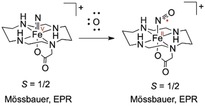

It is of fundamental importance to understand the structure–activity relationship of terminal metal nitrides (N^3−^) because of their involvement in N_2_ reduction^1^ and NH_3_ oxidation processes.^2^ Among all transition metals, iron nitrides attract particular attention owing to their biological relevance.1a, 1b To date, a plethora of iron nitrides in various iron oxidation states have been synthesized, all featuring either tetragonal or trigonal symmetry.^3^ Because most trigonal iron(IV/V) nitrides are isolable, presently the majority of reactivity investigations focus on these species, which indeed exhibit diversified reactivity.^3^, ^4^ For instance, treating such complexes with reducing agents was found to afford ammonia,^5^ and they are typically capable of initiating nitrogen atom transfer to a range of nucleophiles.5c, 6 In contrast, all tetragonal iron(V/VI) nitrides are highly unstable and have to be prepared invariably under cryogenic conditions.^3^ For example, in fluid solutions, [Fe^V^N(cyclam‐ac)]^+^ (**2**, cyclam‐ac^−^=1,4,8,11‐tetraazacyclotetradecane‐1‐acetate anion) undergoes facile dimerization to eventually release N_2_.^7^ The nitrido ligands of tetragonal low‐spin iron(V) nitrides feature considerable radical character, because their singly occupied molecular orbital (SOMO) is one of the two Fe−N π* molecular orbitals (MOs) formed by the antibonding combinations of the Fe d_*xz*/*yz*_ and N p_*x*/*y*_ atomic orbitals (Scheme 1).^8^ In contrast, the SOMO of the corresponding trigonal species is either of the Fe d${}_{x{}^{2}-y{}^{2}}$ or the Fe d_*xy*_ based MOs, both perpendicular to the Fe−N interaction.^9^ Consequently, trigonal iron(V) nitrides are often more stable than their tetragonal analogues. The fleeting nature of the latter poses a challenge to explore their reactivity, and mass spectrometry^10^ or time‐resolved spectroscopy has to be relied upon.^11^ Using these methods, tetragonal iron nitrides were shown to activate C−H and C=C bonds of organic substrates,^10^, ^11^ similar to their trigonal congeners.^12^

**Scheme 1:**
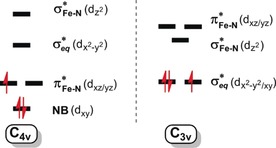
Qualitative orbital splitting pattern for iron(V) nitrides (NB=non‐bonding).

Complex **2** having an *S*=1/2 ground state^13^ exhibits an unusual electron paramagnetic resonance (EPR) spectrum with a slightly asymmetric zero‐crossing derivative signal around *g*_⊥_=1.7 and a broad negative peak at *g*_∥_=1.^8^ Recently, these highly anisotropic *g* values were shown to be a unique signature of tetragonal low‐spin iron(V) nitrides.^8^ Thus, EPR can be used as a sensitive and efficient tool to detect such transient intermediates, which opens up a new way to probe their chemical activity. Herein, we present in situ spectroscopic investigations of oxygenation of **2** to yield the corresponding nitrosyl species (Scheme 2 b). Note that chemisorbed nitrogen atoms on metal surfaces are key intermediates of selective NH_3_ oxidation for industrial NO production.^2^ To the best of our knowledge, conversion of an open‐shell metal nitride into a metal nitrosyl is unprecedented. Only oxygenation of diamagnetic 4d (ruthenium(IV/VI)^14^) and 5d (osmium(VI)^15^ and iridium(III/V)^16^) metal nitrides has been reported (Scheme 2 a), and, more importantly, the underlying mechanism remains poorly understood.

**Scheme 2:**
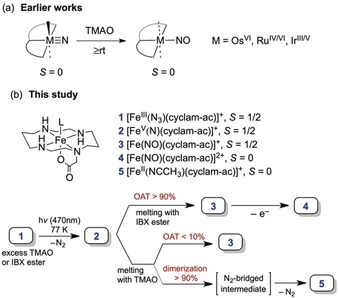
Oxygenation of terminal metal nitrides.

Complex **2** was generated by bulk photolysis of [Fe^III^(N_3_)(cyclam‐ac)](PF_6_) (**1**) in frozen solutions with a yield of almost 100 %, as revealed by EPR investigations (Figure 1 a).^8^ Upon thawing, **2** was found to decay rapidly via dimerization.^7^ To intercept this pathway, a large excess of oxygen atom transfer (OAT) agents was mixed with **1** prior to freezing and illumination. During photolysis, the formation of **2** and its reaction with the OAT reactant in the solid matrix were monitored by repeated spectroscopic measurements of the frozen samples.

**Figure 1:**
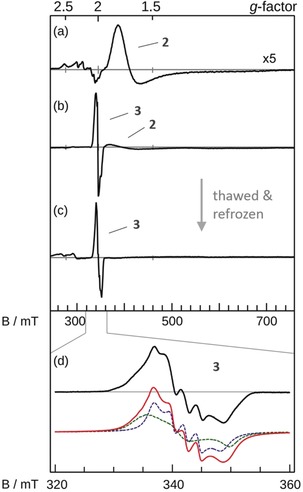
X‐band EPR spectra of a) **2** at 10 K generated without IBX‐ester; b) illuminated **1** with 100 equiv of IBX‐ester; c) the same sample as for (b) after thawing and quick refreezing; d) the zoomed‐in spectrum of (c). In black: experimental spectrum of **3**, red: simulation by superposition of two isomers given in green and blue (For details, see the Supporting Information).

After one‐hour irradiation of the frozen reaction mixture of 1 mm of **1** and 100 equiv of isopropyl 2‐iodoxybenzoate (IBX‐ester) in CH_3_CN, the EPR spectrum revealed emergence of a new nearly isotropic signal centered at *g*≈2 attributed to new species **3**, along with some unreacted **2** (Figure 1 b). Numerical integration showed that the total spin concentration remained unchanged and the relative amount of **3**:**2** was ca. 1:9. Upon thawing, the sample in a cold EtOH bath (−40 °C) and immediately refreezing it, **3** elicited a well‐resolved hyperfine structure, concurrent with complete disappearance of the broad signal of **2** (Figure 1 c, d). Remarkably, the EPR features of **3** match exactly those published for [Fe(NO)(cyclam‐ac)]^+^,^17^ a structurally and spectroscopically well‐characterized *S*=1/2 {Fe‐NO}^7^ complex.^18^ Control experiments demonstrated that IBX‐ester is photostable under the experimental conditions. Spin quantifications revealed that the thawing process led to an approximate ten‐fold decrease in the total spin concentration, and that the maximal yield of **3** is only 10 % relative to **1**. We surmised that once formed, the majority of **3** converted into an EPR‐silent species in the thawed reaction mixture.

To identify the EPR‐silent product, the same reaction was performed with 50 % ^57^Fe‐enriched **1** (5 mm) in CH_3_CN. The 80 K zero‐field Mössbauer spectrum of the frozen photolyzed reaction mixture is dominated by a highly asymmetric and broad doublet known for **2** (Figure 2 a, isomer shift *δ*=0.00 mm s^−1^ and quadrupole splitting |Δ*E*_Q_|=1.72 mm s^−1^).^7^ Unfortunately, the Mössbauer spectrum of **3** (*δ*=0.28 mm s^−1^ and |Δ*E*_Q_|=0.85 mm s^−1^) was also found to have a similar line shape.^17^ Consequently, the overlap of the Mössbauer features of **2** and **3** renders quantification of **3** difficult. Careful simulation analyses indicated that before thawing the sample contained at least 90 % of **2** and less than 10 % of **3** (Figure 2 a; details given in the Supporting Information). Thawing at room temperature and refreezing the sample resulted in a well‐resolved dominating (88 %) quadrupole doublet (Figure 2 b, *δ*=0.00 mm s^−1^ and |Δ*E*_Q_|=1.72 mm s^−1^) attributed to diamagnetic [Fe(NO)(cyclam‐ac)]^2+^ (**4**), the one‐electron oxidized product of **3**, based on its Mössbauer parameters reported earlier.^17^ However, direct generation of **4** from the reaction of **2** with IBX‐ester is unlikely to happen (see below). More importantly, treating independently prepared **3** with a large excess of IBX‐ester was found to afford **4** (Supporting Information, Figure S8). All observations thus suggested that newly formed **3** was oxidized to **4** by unreacted IBX‐ester in the reaction mixture. Hence, the yield of conversion of **2** to **3** must exceed 90 %.

**Figure 2:**
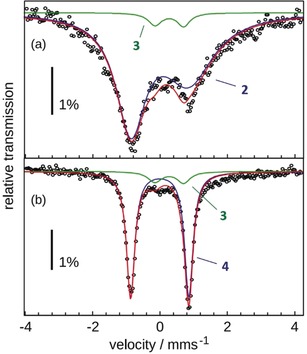
Mössbauer spectra of photolyzed **1** recorded at 80 K in the presence of 100 equiv of IBX‐ester, a) before (**2**: blue; **3**: green) and b) after thawing the sample (**3**: green; **4**: blue). The simulated subspectrum of **3** accounts for 5 % and 12 % of the total iron content in (a) and (b), respectively.

The IR spectrum of the final product generated by the same reaction using ^15^N–^14^N–^14^N^−^ as the starting material showed two bands at 1901 and 1864 cm^−1^ with nearly identical intensity (Figure 3 a). In comparison with the N−O stretching frequency (1903 cm^−1^) reported for **4**‐^14^NO,^17^ the two features can be unambiguously assigned to **4**‐^14^NO and **4**‐^15^NO, respectively, because the measured ^14^N/^15^N isotope shift of 37 cm^−1^ is in good agreement with that (35 cm^−1^) predicted by the harmonic oscillator approximation. The ESI‐MS spectrum of the reaction product also revealed that the overlapping isotope patterns of **4**‐^4^NO and **4**‐^15^NO have similar intensity (Figure 3 b). These findings demonstrated that the N atom in the NO ligand of **3** and **4** originates from the azido ligand of **1**.

**Figure 3:**
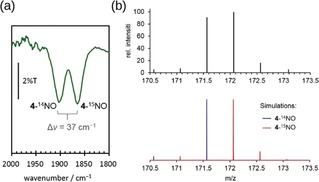
a) IR and b) ESI‐MS spectra of the final product generated by the reaction of ^14^N/^15^N mix‐labeled **2** with IBX‐ester.

We also examined the reaction of 5 mm of **1** with 100 equiv of trimethylamine oxide (TMAO) in a frozen CH_3_CN/MeOH (1:1) solution. The reaction indeed generates **3** albeit with a much lower yield (Scheme 2 b, for details, see the Supporting Information). Specifically, EPR and Mössbauer measurements suggested that the yield of **3** is about 2 % relative to **1**, and the main product (93 %) is [Fe^II^(CH_3_CN)(cyclam‐ac)]^+^ (**5**),^7^ thereby indicating that the OAT process with TMAO cannot compete with the dimerization of **2**.

To gain deep insight into the reaction mechanism, we performed detailed computational investigations. The reactions of **2** with IBX‐ester and TMAO to generate **3** were predicted to be highly exothermic by 74.1 and 62.2 kcal mol^−1^, respectively. In contrast, the theoretical results revealed that the reaction of **2** with IBX‐ester to yield **4** possesses a much lower driving force (−15.7 kcal mol^−1^). Kinetically, the computed potential energy surface (PES) for the OAT process with IBX‐ester is always downhill (Supporting Information, Figure S9). Both factors thus render the direct generation of **4** from the reaction of **2** with IBX‐ester improbable to occur. Unlike the reaction with IBX‐ester, the transformation with TMAO is nearly barrierless, yet with a plateau in the PES upon TMAO approaching the (FeN)^2+^ core (Figure 4 a). Note that the dimerization process of **2** features an even higher driving force (ca. −120 kcal mol^−1^), and the calculated PES shows a constant decrease in energy without a discernible barrier.^7^ Therefore, considering the reaction thermodynamics and kinetics, the OAT reaction with IBX‐ester has a higher probability than that with TMAO to compete with the self‐decay of **2**, when a large excess of the chosen OAT agent was applied. This is why the different outcome was found for the reaction with TMAO and IBX‐ester.

**Figure 4:**
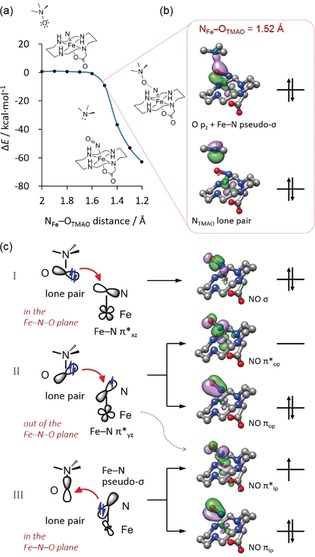
a) Potential energy surface of the reaction of **2** with TMAO computed by a relaxed surface scan of the N_Fe_−O_TMAO_ distance; b) representative orbitals at the N−O distance of 1.52 Å; c) orbital interactions to form the N−O multiple bonds of **3**.

As shown in Figure 4 c, as the O atom of TMAO approaches the nitrido ligand of **2**, the O 2p based lone pair of TMAO in the Fe−N⋅⋅⋅O_TAMO_ plane overlaps the unoccupied Fe−N π*_xz_ MO, which forms the NO σ bonding MO in the end. The other lone pair perpendicular to the Fe−N⋅⋅⋅O_TMAO_ plane interacts with the singly populated Fe−N π*_yz_ orbital to generate the N−O π_op_ and π*_op_ MOs. When the N_Fe_⋅⋅⋅O_TMAO_ distance is shortened to 1.52 Å, the O−N σ bond (2.627 Å) in TMAO is essentially broken, thereby resulting in a doubly occupied N 2p based orbital and a vacant O 2p centered orbital (Figure 4 b). The former finally evolves to the lone pair of trimethylamine, and the latter interacts with the doubly occupied Fe−N pseudo σ‐bonding orbital, leading to the in‐plane NO π bond. The out‐of‐plane π interaction involves three electrons, but the unpaired electron in **2** resides in the in‐plane NO π*_ip_ MO rather than NO π*_op_ as would be expected. As borne out from Figure 4 c, the NO π*_ip_ centered MO is stabilized by the bonding interaction between the NO π*_ip_ and Fe d${}_{z{}^{2}}$ fragment orbitals, whereas the NO π*_ip_ based MO is an antibonding combination with respect to the Fe−NO interaction. Therefore, NO π*_ip_ MO has lower energy than NO π*_op_.^17^

In summary, during the reaction of **2** to **3**, the OAT agent essentially donates a closed‐shell singlet O atom to **2**, and the (FeN)^2+^ unit functions not only as an electron acceptor but also an electron donor. Therefore, the reaction does not follow a radical‐coupling mechanism as postulated for the N_2_ expulsion,^7^, ^16^ but reveals the ambiphilic nature of **2** found for related diamagnetic metal nitrides.^19^ Consequently, the synergetic orbital interactions accompanied by the mutual electron transfer between **2** and the O‐atom donor build up N−O multiple bonds in a concerted yet asynchronous way without an intervening intermediate. On the basis of the above analysis, the oxygenation of closed‐shell metal nitrides should follow a similar mechanism. This work enriches the already diversified reactivity of high‐valent iron nitrides and provides a different viable strategy for constructing multiple bonds for diatomic molecules.

## Conflict of interest

The authors declare no conflict of interest.

## Supporting information

As a service to our authors and readers, this journal provides supporting information supplied by the authors. Such materials are peer reviewed and may be re‐organized for online delivery, but are not copy‐edited or typeset. Technical support issues arising from supporting information (other than missing files) should be addressed to the authors.

Supplementary
