# Supplementary material for: Conversion of a Fleeting Open‐Shell Iron Nitride into an Iron Nitrosyl
Source: Angew Chem Int Ed Engl. 2019 Oct 22;58(49):17589–93. doi: 10.1002/anie.201908689 (PMC6899486; doi:10.1002/anie.201908689)
Supplement: Supplementary file 1 — Supplementary [file ANIE-58-17589-s001.pdf]

Angewandte Chemie
Eine Zeitschrift der Gesellschaft Deutscher Chemiker

Supporting Information

Conversion of a Fleeting Open-Shell Iron Nitride into an Iron Nitrosyl

Hao-Ching Chang, Yen-Hao Lin, Christophe Werlé, Frank Neese, Way-Zen Lee, Eckhard Bill,* and Shengfa Ye**

anie_201908689_sm_miscellaneous_information.pdf

Experimental Method

Isopropyl 2-iodoxybenzoate (IBX-ester)[s1] and $[Fe^{III}(N_3)(cyclam\text{-}ac)](PF_6)$ (**1**)[s2] were prepared as described in literature. When needed, ^{15}N-labelled NaN_3 or ^{57}Fe metal were utilized in synthesis. Trimethylamine *N*-oxide (TMAO) was used as it purchased from vendor.

Complex **1** was dissolved in CH_3CN and mixed with stock solutions of a large excess of OAT reagents, IBX ester in CH_3CN or TMAO in MeOH, at –40 ºC to give reaction mixtures. For EPR measurements, the concentration of complex **1** in the mixture is 1 mM. The solutions were loaded into standard 4 mm quartz EPR tubes and frozen in liquid nitrogen inside a quartz dewar prior to illumination by a LUXEON III Star LED lamp (dominant wavelength of 470 nm). One-hour irradiation was enough to completely convert **1** to **2** in this setup. Thawing EPR samples were performed in a cold EtOH bath (ca. −40 ºC). The tubes were warmed up as the frozen solution got just melted, and then the fluid solution was immediately refrozen by liquid nitrogen. The entire thawing process lasted less than one minute. *WARNING! The pressure should be released to avoid explosion of the quartz tube if the sample was not flame-sealed under vacuum.* The EPR samples were then measured on a continuous-wave Bruker E500 ELEXSYS spectrometer equipped with the Bruker dual-mode cavity (ER4116DM), an Oxford Instruments helium flow cryostat (ESR 900), and a high-sensitivity Super-X microwave bridge (Bruker ER-049X). The magnetic field controller (ER032T) was calibrated by using a Bruker NMR field probe (ER035M). The spectra were generally recorded at 9.63 GHz resonant frequency with 0.20 mW microwave power and 0.75 mT/100 kHz modulation.

To prepare Mössbauer samples, the reaction mixture containing 5 mM 50% ^{57}Fe-enriched **1** was frozen in liquid nitrogen and grounded into fine powder for the photolysis. Due to the different preparation procedure, four-hour illumination with periodic stirring of the powder was required in this setup. The illuminated frozen-solution powder was then loaded into sample cups made of DELRIN (ca. 0.7 mL), capped and mounted on a sample rod chilled with liquid nitrogen. Thawing Mössbauer samples was performed under air in a chilled container (e.g. dewar), and lasted less than 1 minute prior to refreezing with liquid nitrogen. ^{57}Fe Mössbauer spectra were recorded by a conventional Mössbauer spectrometer with alternating constant acceleration of the γ-source (^{57}Co/Rh, 1.8 GBq). The spectra have a minimum line-width of 0.24 mm/s (full width at half-height). The sample cavity was maintained at 80 K by an Oxford Instruments Variox cryostat. Isomer shifts are quoted relative to iron metal at 300 K. Spectroscopic simulations were performed by using our own routines, with *esimX* or *mf*.[s3]

FTIR (Fourier transform infrared) spectra were collected with a Thermo Scientific Nicolet iS50 spectrometer in attenuated total reflection (ATR) mode with a resolution of 4 cm^{-1} at room temperature. ESI–MS spectra were obtained by using high resolution mass spectrometry FT-ICR (Fourier transform ion cyclotron resonance) coupling with ESI (electrospray ionization) (+) at the mass range of 50 to 600 m/z.

As shown in Figure S1, the EPR spectrum of the illuminated reaction mixture before thawing exhibits an unresolved sharp peak of **3** together with some residual broad signal of **2**. By warming up the frozen sample in a cold bath and refreezing the sample as it melted, the signal of **2** depleted and the pattern of the sharp peak was simplified, shown as Figure S1. As elaborated elsewhere,[S4] the crystal structure of **3** shows that the NO ligand is disordered arising from the different Fe–NO angles, and the EPR spectrum of independently prepared **3** reveals at least two isomers with distinct ^{14}N hyperfine matrices. Therefore, we tentatively attributed the unresolved hyperfine structures to the microheterogeneity of newly formed **3** in the frozen solution. In fact, these two isomers with the same ratio were also needed for simulating the well-resolved EPR spectrum (Figures 1d and S1b). The signal at 250–300 mT in Figure 1c in the main text is the same low-spin Fe(III) impurity as that shown in Figure S5. Its yield is only ~5% with respect to **1**, and is resulted presumably from degradation of **1** or side reactions via **2**, as we found by mixing TMAO with **1** at −40 ºC in pure CH_3CN.

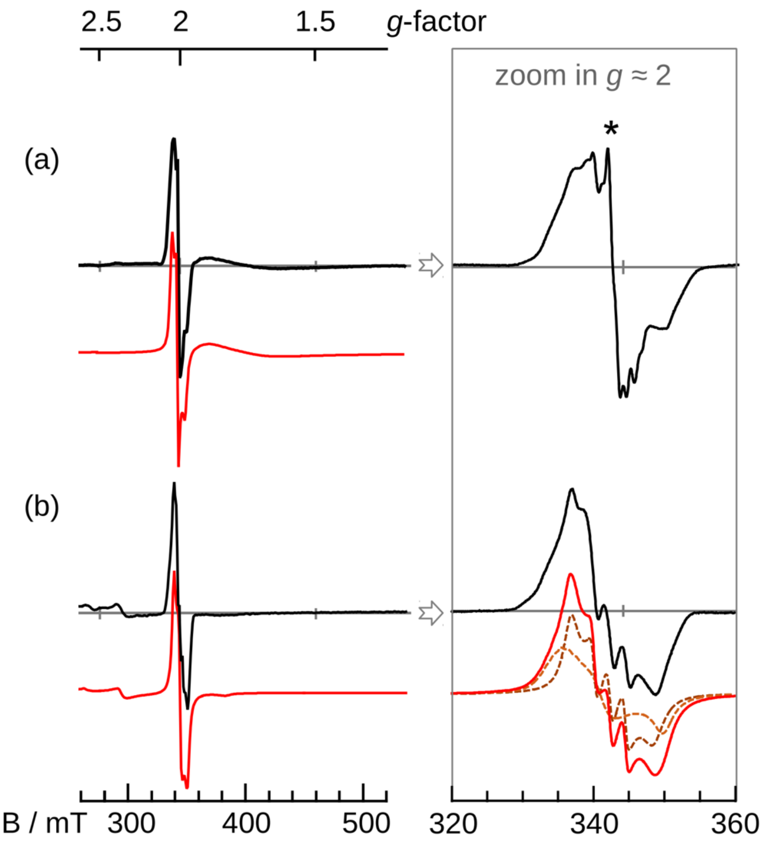

Figure S1. 10 K X-band EPR spectra of illuminated **1** with 100 equiv IBX ester (a); thawing and quickly refreezing the same sample (b). Frequency 9.63 GHz, power 0.20 mW power, modulation 0.55

mT/100kHz. Simulation parameters of isomer I: g (2.043, 2.011, 1.976), W_f 7 G with W_{xyz} (13, 0, 20 /G) A_N (2, 75, 30 /MHz), weight 24%; those of isomer II: g (2.052, 2.019, 1.968), W_f 8 G with W_{xyz} (5, 24, 18 /G) A_N (54, 63, 54 /MHz), weight 76%. W_f is the frequency-dependent linewidth, and $W_{x,y,z}$ are field-dependent linewidths of the $g_{x,y,z}$ components, respectively.

As depicted in Figure 2a, the Mössbauer spectrum of the frozen reaction mixture is dominated by the characteristic signal of **2**, an highly asymmetric and rather broad quadrupole doublet with linewidth Γ ~ 1.0 mm/s.[S4] In fact, our earlier work showed that **3** in solid state also exhibits a slightly asymmetric and broad doublet (Γ ~ 0.5 mm/s).[S5] The Mössbauer feature of **3** in frozen solutions should be even broader than that found in the solid state due to slower electron-spin relaxation. As a consequence, the Mössbauer features of **3** and **2** are heavily overlapped, it is difficult to accurately quantify the amount of **3** in the Mössbauer samples. Nevertheless, after repeated attempts to deconvolute the Mössbauer spectra with the fixed Mössbauer parameters including the linewidth (1 and 0.5 mm/s for **2** and **3**, respectively) (Table S1), we can conclude that the sample before thawing contains >90% of **2** and <10% of **3**. For example, if the sample consists of 12% of **3 (**Figure S2), the simulation is unacceptable.

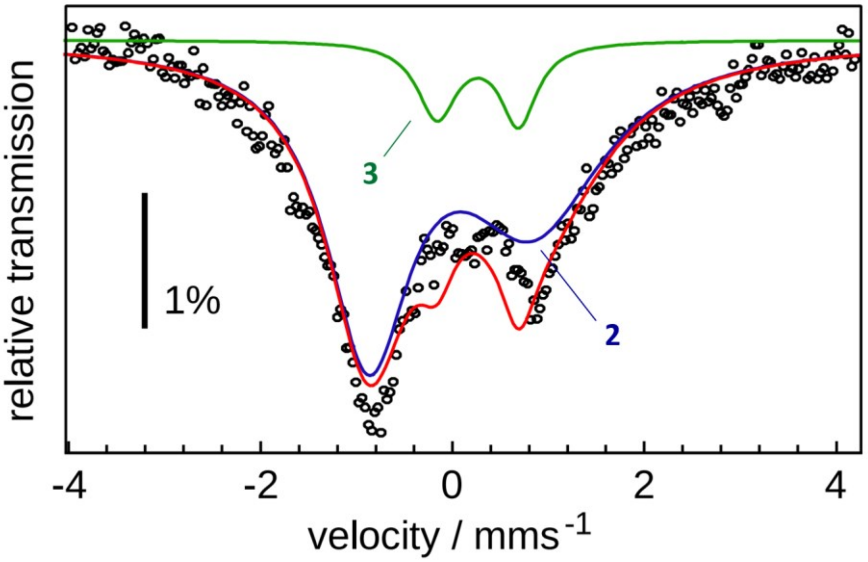

Figure S2. Mössbauer spectrum of photolyzed **1** recorded at 80 K with 100 equiv IBX ester before thawing. The green trace represents 12% of **3** with respect to the total iron content. To mimic not perfectly fast spin relaxation for a half-integer spin species, such as **2**, asymmetric broadening is introduced to simulate the zero-field Mössbauer spectra, i.e. the two lines have the same intensity but different linewidth.

^{15}N-enriched sodium azide ($^{15}N–^{14}N–^{14}N^{-}$) was utilized to synthesize **1** with isotope-labelled ligand, so photolysis produces 1:1 ^{14}N/^{15}N mixed-labelled complex **2**. Reactions of labelled **1** and 100 equiv IBX ester were performed in a way similar to that used to prepare the Mössbauer samples, ca. 1 mL frozen powder was illuminated for three hours prior to thawing. On benchtop the solution was then diluted with 3 mL of water and extracted several times with CH_2Cl_2 to remove most unreacted IBX ester. The remaining aqueous solution was evaporated; the residual was scratched off for ATR-IR measurements. As shown in Figure S3, two distinct peaks at 1901 and 1864 cm^{-1} were detected and can be safely assigned as **4**-^{14}NO and **4**-^{15}NO, according to the published data.[S5] Notably, without the extraction both signals are buried by the absorption of excess IBX ester. Despite this, the N-O stretching vibration for **3**-^{14}NO (1607 cm^{-1})[S5] and **3**-^{15}NO (1578 cm^{-1}) remain completely obscured by the intense absorption of IBX ester.

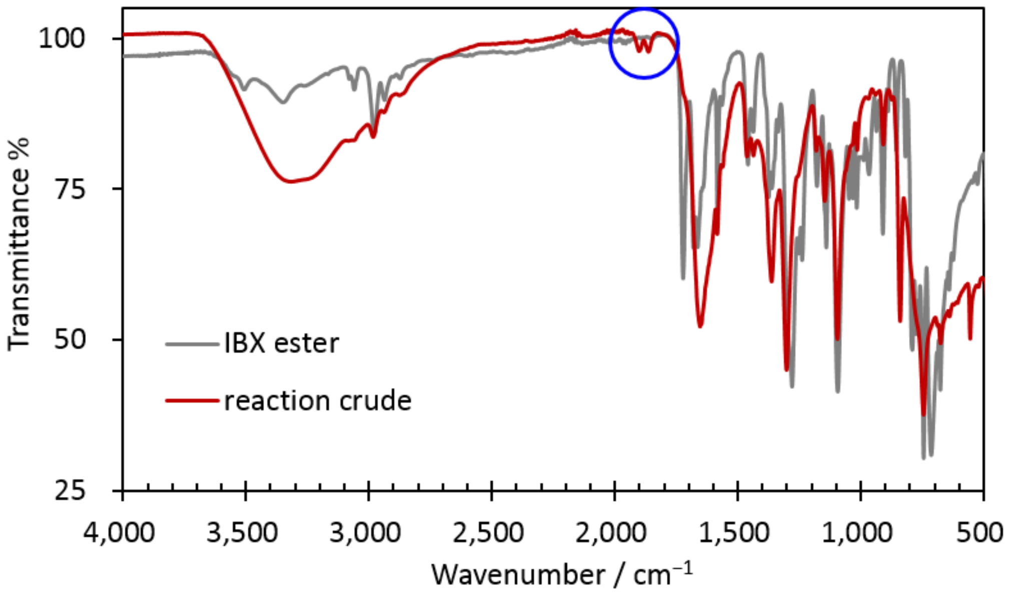

Figure S3. ATR-IR spectrum of 50% ^{15}N-labelled **4** (red trace) and excess IBX ester reagent (grey trace). The sample powder were measured at ambient condition.

^{15}N-labelled samples were also used for ESI–MS spectrometric measurements. The reaction crude was extracted with the aforementioned method. As shown in Figure S4, ionized fragments of the final product were identified and the isotope patterns attributed to two isomers, [**4**-^{14}NO]$^{2+}$ and [**4**-^{15}NO]$^{2+}$, have similar intensity. Corresponding simulations are summarized in Figure 3b.

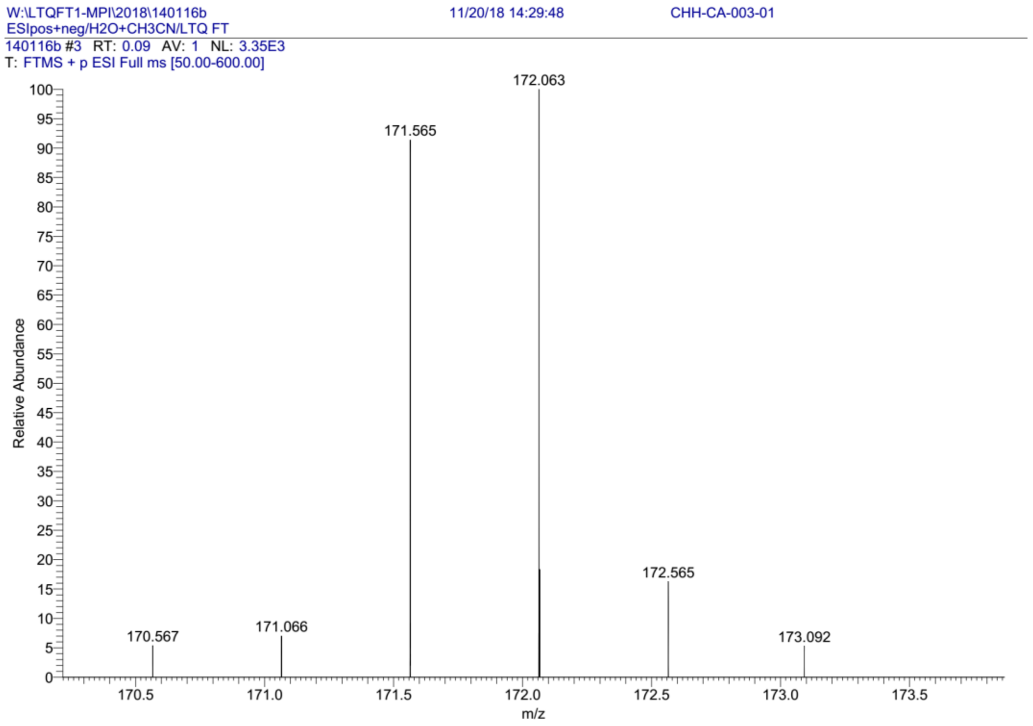

Figure S4. ESI–MS spectrum of the final product generated by the reaction between IBX ester and $^{14/15}N$ mix-labelled **2**. Simulations for the two isotopic ions are shown in Figure 3b.

Figure S5 shows formation of a low-spin ferric complex (**A**) by mixing TMAO with **1** at –40 ºC in pure CH_3CN. The spectrum (black trace) reveals the new species having *g*–values of 2.49, 2.21, 1.88 (red dashed line), different from those of **1** (2.60, 2.29, 1.82). The reaction to form **A** in pure CH_3CN proceeds very fast even at –40 ºC; consequently, the spectrum shown in Figure S5 is mainly dominated by the feature of **A**. To avoid this side reaction, further reactions with TMAO were therefore performed in MeOH/CH_3CN mixed solvent, whereby **1** is largely intact during the mixing process (Figure S6a). But species **A** is still generated in the following photolysis (Figure S6b).

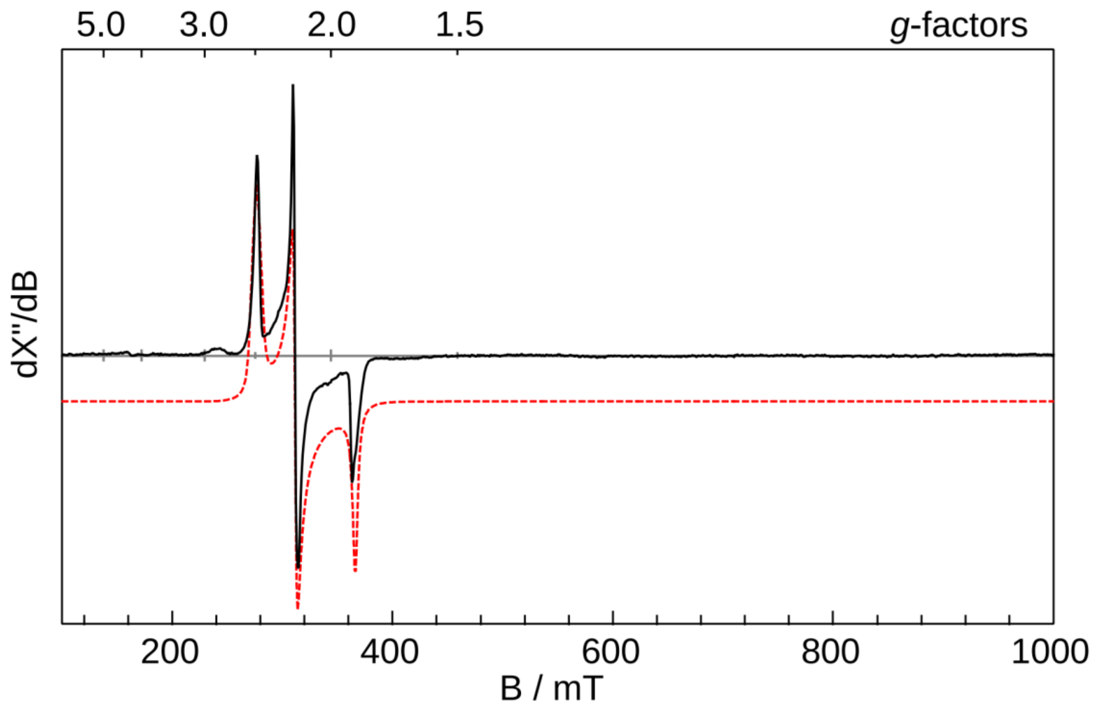

Figure S5. 10 K X-band EPR spectrum of **1** with excess TMAO in CH_3CN. Frequency 9.63 GHz, power 0.20 mW, modulation 0.75 mT/100kHz. The reaction mixture was prepared with pure CH_3CN, by mixing stock solutions of **1** and TMAO in cold bath and frozen rapidly. Simulation parameters: *g* (2.490, 2.210, 1.880), frequency-dependent linewidth W_f 30 G.

Initially the EPR spectrum of the reaction mixture is dominated by the signal of **1** (92%) with some species **A** (8%, Figure S6a). After the illumination (Figure S6b), the spectrum is composed of three major species: Fe^{V}-nitrido complex **2** (90%), ferric species **A** (8.5%), and **3** (1.5%) that is characterized by a sharp peak centered at $g \approx 2$. Note that in Figure S6b the overall spin concentration remains nearly unchanged, implying that the generation of **2** is quite smoothly by the photolysis.

However, after thawing and quickly refreezing the sample, the total spin concentration drops by approximate one order of magnitude. This observation indicates that the majority of the paramagnetic species has converted into diamagnetic ones. The remaining signals were deconvoluted into three major components: complex **3** (rel. 15%), species **A** (rel. 54%), and another low-spin ferric species **B** (rel. 31%, g = 2.60, 2.29, 1.81). In the thawed sample, **3** elicits well resolved hyperfine pattern as shown in Figure S1b. Therefore, all findings confirm the formation of **3** from the reaction of **2** with TMAO albeit in a trace amount, which is due to the facile N–N coupling and/or the existence of several side-reaction channels.

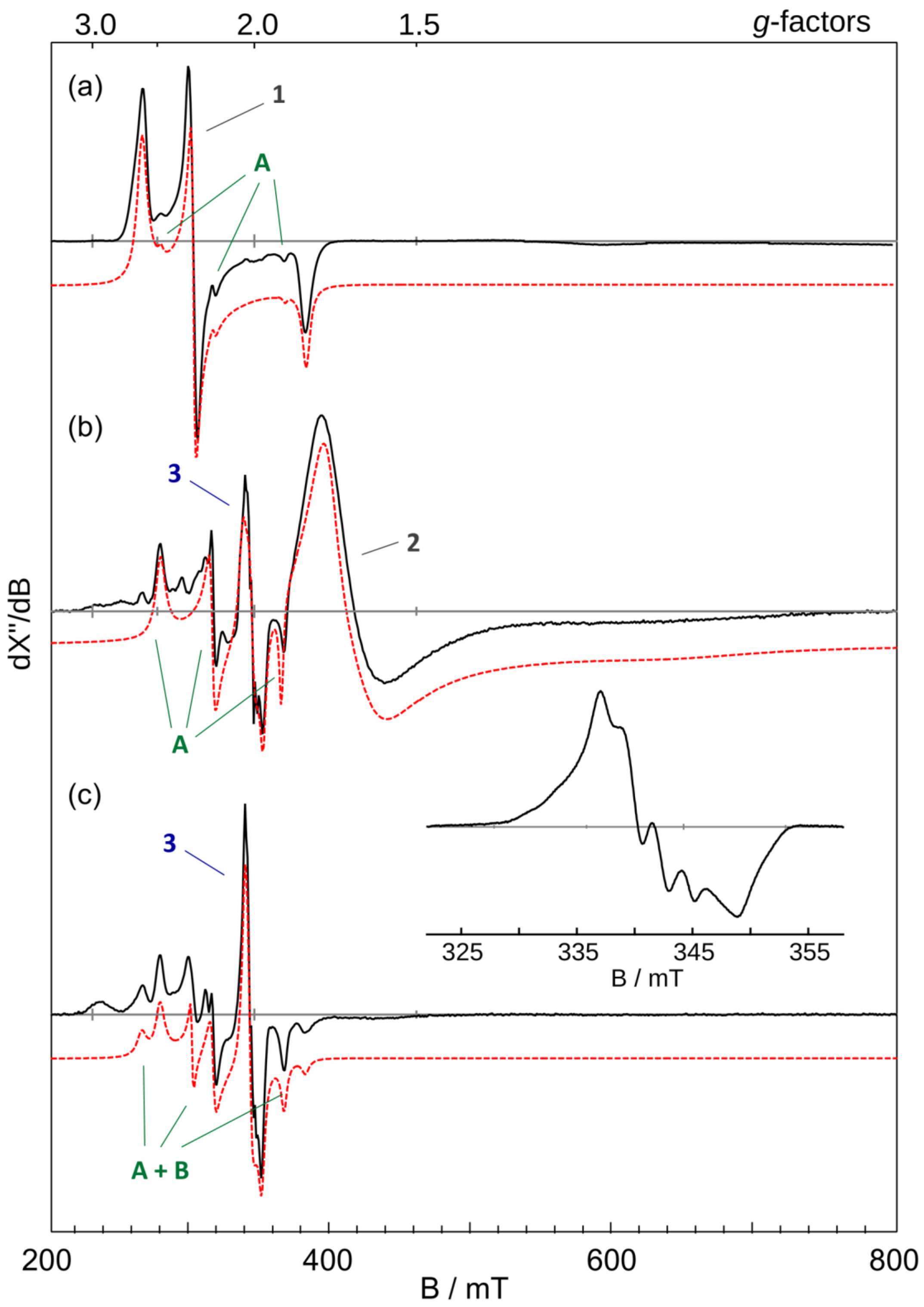

Figure S6. 10 K X-band EPR spectra of 2.5 mM **1** with 100 equiv TMAO in MeOH/CH_3CN 1:1 (v/v): (a) the reaction mixture; (b) the illuminated sample; and (c) the thawed and refrozen sample. Frequency 9.63 GHz, power 0.20 mW, modulation 0.75 mT/100kHz. The sample was prepared aforementioned

protocol except for the oxygenating reagent and solvents employed. Simulations are shown as red dashed lines. The inset of (c) is the zoomed-in spectrum for the sharp signal of $g \approx 2$. Simulation parameters: (a) g (2.605, 2.289, 1.808), W_f 25 G with W_{xyz} (45, 0, 26 /G), weight 92%; g (2.480, 2.182, 1.882), W_f 20 G with W_{xyz} (15, 0, 0 /G), weight 8%; (b) g (1.73, 1.67, 1.07), W_f 60 G with W_{xyz} (120, 310, 960 /G), weight 90%; g (2.050, 2.010, 1.965), W_f 15 G with W_{xyz} (35, 30, 25 /G), weight 1.5%; (c) g (2.482, 2.186, 1.885), W_f 26 G with W_{xyz} (38, 10, 0 /G), weight 54%; g (2.605, 2.295, 1.810), W_f 15 G with W_{xyz} (45, 0, 30 /G), weight 31%; g (2.040, 2.018, 1.970), W_f 30 G, weight 15%. W_f is the frequency-dependent linewidth, and $W_{x,y,z}$ are field-dependent linewidths of the $g_{x,y,z}$ components, respectively.

Simulations (red traces) revealed three major iron species in the frozen sample: **2** (73% blue dashed line), **3** (6% green dashed line) and **5** (21% orange dashed line). Formation of the large amount of **5** is likely due to the prolonged photolysis required for preparing the Mössbauer sample. Upon thawing the sample, most complex **2** converted to **5** (93%), resulting from the facile N–N coupling reaction. Parameters are summarized in Table S1. Note that Figure S6b does not allow us to accurately quantify the amount of by-products **A** and **B** detected by EPR, basically because of their unknown Mössbauer parameters.

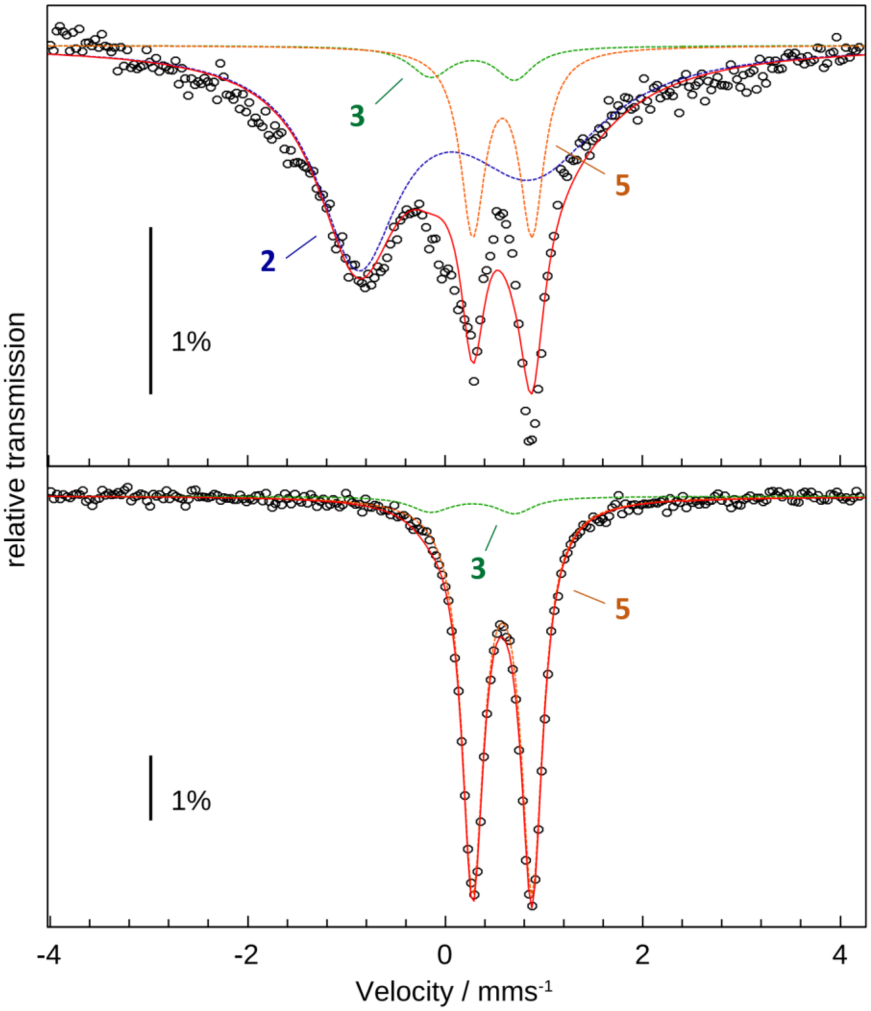

Figure S7. 80 K zero-field Mössbauer spectra of photolyzed 5 mM **1** in the presence of 100 equiv TMAO, before (top) and after (bottom) melting.

Complex **3** was prepared independently according to the literature method,[s5] and reacted with a large excess of IBX-ester in CH_3CN in the presence and absence of air. In both conditions, **3** was found to be oxidized to **4** as confirmed by infrared spectroscopic measurements (Figure S8). Specifically, upon treating **3** with IBX-ester aerobically and anaerobically, the characteristic N–O (1611 cm^{-1}) and C=O (1661 cm^{-1}) stretching vibrations of **3** disappeared, and the N–O (1903 cm^{-1}) and C=O (1713 cm^{-1}) bands of **4** emerged.

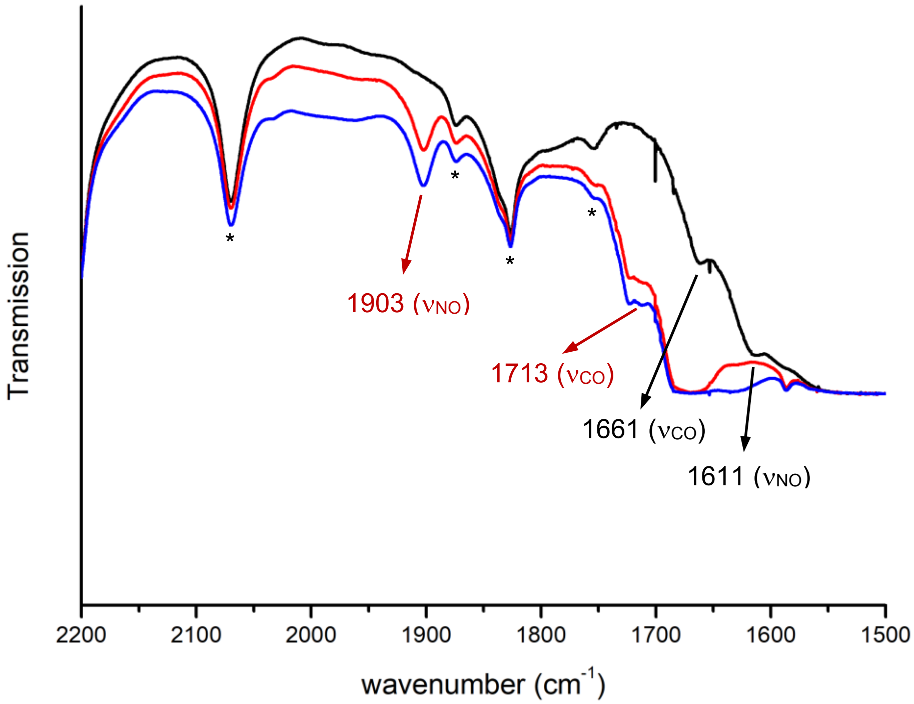

Figure S8. Infrared spectra of **3** (black trace), and the reaction solutions of **3** with a large excess of IBX-ester in CH_3CN aerobically (blue trace) and anaerobically (red trace). Asterisks denote the peaks of impurities existed in **3**.

Table S1. Mössbauer parameters of relevant iron complexes

	δ / mms^{-1}	$\|\Delta E_Q\|$ / mms^{-1}	Linewidth / mms^{-1}	ref.
$[Fe^{III}(N_3)(cyclam\text{-}ac)]^+$ (**1**)	+0.27	2.48	0.30	s4
$[Fe^{V}N(cyclam\text{-}ac)]^+$ (**2**)	–0.01	1.58	1.00	s4
$[\{Fe(NO)\}^7(cyclam\text{-}ac)]^+$ (**3**)	+0.28	0.86	0.50	s5
$[\{Fe(NO)\}^6(cyclam\text{-}ac)]^+$ (**4**)	+0.02	1.71	0.28	s5
$[Fe^{II}(CH_3CN)(cyclam\text{-}ac)]^+$ (**5**)	+0.57	0.59	0.28	s4
photolyzed **1** with IBX ester	0.00[a]	1.76 (>90%)[a]	1.00	this work
	+0.28	0.85 (<10%)	0.50	
thawed sample with IBX ester	0.00	1.72 (88%)	1.00	this work
	+0.28	0.85 (12%)	0.50	
photolyzed **1** with TMAO	0.00[a]	1.76 (73%)[a]	1.00	this work
	+0.58	0.60 (21%)	0.28	
	+0.28	0.85 (6%)	0.50	
thawed sample with TMAO	+0.58	0.60 (93%)	1.00	this work
	+0.28	0.85 (7%)	0.28	

[a] The quadrupole doublet of **2** becomes more asymmetric and broader in frozen solutions.

Computational set-up

All calculations were performed by ORCA 4.0 quantum chemical program.[s6] The BP86 functional[s7] in conjunction with the approximation of resolution of the identity (RI) was used for geometry optimizations,[s8] for which def2-SVP basis sets and def2/J auxiliary basis sets were employed.[s9] Solvation effects were taken in account by using the conductor-like polarizable continuum model (CPCM)[s10] with CH_3CN being the solvent. To model the oxygen atom transfer reaction, a relaxed surface scan, wherein the target N–O bond distance was systematically shortened, was performed starting from **2**-IBX ester and **2**-TMAO adducts. For each points, the electronic energy shown in Figure 4a was calculated by the hybrid B3LYP functional.[s11]

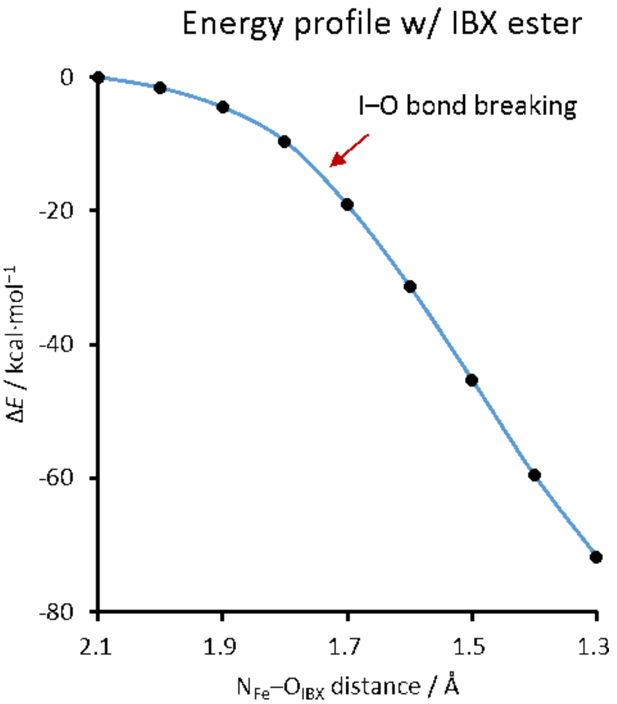

Figure S9. The potential energy surface of the reaction with IBX ester computed by a relaxed surface scan of the N_{Fe}–$O_{IBX\ ester}$ distance.

References

s1. V. V. Zhdankin, A. Y. Koposov, D. N. Litvinov, M. J. Ferguson, R. McDonald, T. Luu, R. R. Tykwinski, *J. Org. Chem.* **2005**, *70*, 6484–6491.

s2. C. A. Grapperhaus, B. Mienert, E. Bill, T. Weyhermüller, K. Wieghardt, *Inorg. Chem.* **2000**, *39*, 5306–5317.

s3. Spectroscopic analysis package developed by Dr. E. Bill (request via eckhard.bill@cec.mpg.de).

s4. O. Krahe, E. Bill, F. Neese, *Angew. Chem.* **2014**, *126*, 8872–8876; *Angew. Chem. Int. Ed.* **2014**, *53*, 8727–8731.

s5. R. G. Serres, C. A. Grapperhaus, E. Bothe, E. Bill, T. Weyhermüller, F. Neese, K. Wieghardt, *J. Am. Chem. Soc.* **2004**, *126*, 5138–5153.

s6. F. Neese, *WIREs Comput. Mol. Sci.* **2018**, *8*:e1327.

s7. (a) A. D. Becke, *Phys. Rev. A* **1988**, *38*, 3098–3100; (b) J. P. Perdew, *Phys. Rev. B* **1986**, *33*, 8822–8824; (c) J. P. Perdew, *Phys. Rev. B* **1986**, *34*, 7406.

s8. O. Vahtras, J. Almlöf, M. W. Feyereisen, *Chem. Phys. Lett.* **1993**, *213*, 514–518.

s9. (a) A. Schafer, H. Horn, R. Ahlrichs, *J. Chem. Phys.* **1992**, *97*, 2571–2577; (b) F. Weigend, R. Ahlrichs, *Phys. Chem. Chem. Phys.* **2005**, *7*, 3297–3305.

s10. J. Tomasi, B. Mennucci, R. Cammi, *Chem. Rev.* **2005**, *105*, 2999–3094.

s11. (a) A. D. *J. Chem. Phys.* **1993**, *98*, 5648–5652; (b) C. Lee, W. Yang, R. G. Parr, *Phys. Rev. B* **1988**, *37*, 785–789.
